# Ant schnapps for health and pleasure: the use of *Formica rufa* L. (Hymenoptera: Formicidae) to flavour aquavit

**DOI:** 10.1186/s13002-019-0347-7

**Published:** 2019-12-19

**Authors:** Ingvar Svanberg, Åsa Berggren

**Affiliations:** 1 Institute for Russian and Eurasian Studies, Uppsala University, Box 514, SE-751 20 Uppsala, Sweden; 2 Department of Ecology, Swedish University of Agricultural Sciences, Box 7070, SE-750 07 Uppsala, Sweden

**Keywords:** Alcoholic beverages, Ethnobiology, Ethnoenthomology, Folk remedy, Insects as food, Future drinks, Local knowledge

## Abstract

**Background:**

The relationship between humans and insects goes long back and is important. Insects provide a multitude of ecosystem services for humans, e g. by pollinating crops and decomposing matter. Our current knowledge about the cultural ecosystem services that insects provide is limited and not much examined.

**Method:**

Scattered ethnographical descriptions and folklore records from pre-modern Sweden and other Scandinavian countries give us insights into local knowledge and use of insects among the peasantry in various parts of the country. These data have been analysed and critically reviewed. Source pluralism has been used as a method.

**Results:**

The mound-building red wood ant, *Formica rufa* L., is one of the species that were used in Sweden for their healing properties. It was a widespread belief that the formic acid could be used to cure various diseases, especially gout and rheumatism. Both anthills and the ants themselves were used for that purpose. It was also common to flavour distilled liquor (*brännvin*) with ants; a remedy used for medicinal purposes. However, already in the eighteenth century, this was also used as schnapps. The cultural services provided by this species stretches throughout history and still exists today.

**Conclusion:**

While the use of ants in medicine has vanished, the custom of making homemade ant flavoured alcoholic beverage survives in Sweden. Nowadays it is a hobby among people who flavour their own aquavit. It is appreciated as a tasty and interesting drink.

## Introduction

The relationship between humans and their biological environment is complex and change over time [1]. The benefits that humans reap from having insects in their environment are many. This was acknowledged early; in the eighteenth century, Carl Linnaeus stressed in his lectures Collegium diæticum that insects served the human being in multivarious ways [2]. The ecosystem services that insects perform are countless (e.g. by providing feed, supporting decomposition of matter, regulating pests and by having cultural values). While the critical role of pollinators in supporting global food supply has become widely recognised, less known is that insects influence virtually all other ecosystem services including cultural ecosystem services [3, 4]. Cultural ecosystem services are nonmaterial benefits like cognitive development, cultural heritage value, spiritual enrichment, religious inspiration, reflection, recreation and aesthetic experiences [5]. The cultural values that insects provide are numerous, but our knowledge of the cultural, economic and social significance of many invertebrates is still scant. For instance, while insects and insect products are a staple food in many parts of the world [6–8], they have played a relatively small role in the diet of the Swedish people during the last centuries [9]. In the Nordic countries, honey and nectar provided by domestic and feral European honeybees, *Apis mellifera* Linnaeus, 1758, and to a much smaller extent bumblebees, have been the most commonly used insect products. Honey and nectar have been and continue to be sought after for their sweetness [10–12].

Additionally, there are other biocultural domains that are created in the activity contexts between humans and insects [10, 13, 14]. The belief that insects have healing power has been widespread since ancient times [15, 16]. In the professional scholarly medicine, some species of insects were used and could be bought in pharmacies. For instance, in the Stockholm Pharmacopoeia of 1686 [17] and in the Swedish Pharmacopoeia of 1775 [18], several insects or derivates of insects were available: e.g. remedies and tinctures made of the stag beetle, *Lucanus cervus* (L.); Spanish fly, *Lytta vesicatoria* (L.); cochineal, *Dactylopius coccus* Costa; kermes, *Kermes ilicis* (L.); silk worm, *Bombyx mori* L.; European honeybee, *Apis mellifera* L.; red wood ant, *Formica rufa* L.; oak gall wasp, *Cynips quercusfolii* L.; and bedeguar, *Diplolepis rosae* (L.) [19, 20]. Spanish fly and bee products were still available for therapeutic uses in Swedish pharmacies at the end of the nineteenth century [21]. Some of these insects were used in the local folk medicine of the peasantry in the pre-industrial (prior to 1880) Swedish society. In old folklore records, we find cures for different ailments by eating bed bugs, *Cimex lectularius*, and head lice, *Pediculus humanus* ssp. *capitis* [22].

The red forest ant, *Formica rufa* L., has a long tradition of use in remedies in Sweden and neighbouring countries and has been available as oleum (oil) and acid in the pharmacies at least since the seventeenth century [23]. Homemade ant schnapps (Swedish *myrbrännvin)* has also been popular. The variation of traditional use of the red forest ant in Sweden has not been previously examined. By highlighting people’s use of this species through history, we are able to better understand the cultural ecosystem services that this species provides.

## Methods and sources

The objective of this study is to gather information of the use of *Formica rufa* in folk medicine and as a flavouring for schnapps in Sweden. While the former use is now extinct, the latter continues to survive in today’s Sweden. “The past is a different culture”, as anthropologists Krech and Sturtenvant aptly put it, and therefore we need historical methods for studying this practice [24, 25]. Our study uses a qualitative approach to understand the past use of ants in remedies and beverages. There are several kinds of sources that can be used: folk life and folklore records, medicinal literature, topographic literature (including travelogues) and zoological literature. The data that this study is based on come from records in the Institute for Language and Folklore, Uppsala, as well as scattered information found in the ethnographic literature and in travel reports [26]. The interaction between ants and humans is a biocultural domain that has been studied very little so far. Our study will therefore contribute to the field of using wild insects as food and medicine. Due to the various kinds of historical sources, we have adopted what historian Janken Myrdal calls source pluralism as a method, when analysing the data [27].

## The red wood ant

The red wood ant *F. rufa* is distributed across Europe and North America [28]. The species provides ecosystem services as ecosystem engineers and as a predator on pests. The anthills of the red wood ant are usually found in forests; they are conspicuous, dome-shaped mounds often constructed using Scots pine (*Pinus sylvestris*) needles and generally built against tree trunks and stumps. Nest may be isolated or occur in small groups and they usually have many queens, up to hundred or more. The numbers of workers in a population can range from 100,000 to 400,000. The size of the hills reflects the health of the colony [29] and only large nest can thermoregulate independently [30]. The main threat to the species is believed to be fragmentation of its forest habitats [31].

Formic acid (CH_2_O_2_) is used by the red wood ants as a defence towards predators, as an alarm pheromone and potentially to aid in capturing prey [32]. Biologically produced formic acid seems not to adversely affect vertebrates if they are able to control the exposure themselves [33]. Birds use formic acid and similar compounds in a specialised behaviour called “anting” to reduce their ectoparasite load [34]. Acid produced in the chemical industry is known to cause injuries or be fatal at high concentration and doses [35].

## Results and discussion

### The traditional use of the red wood ant

**Fig. 1:**
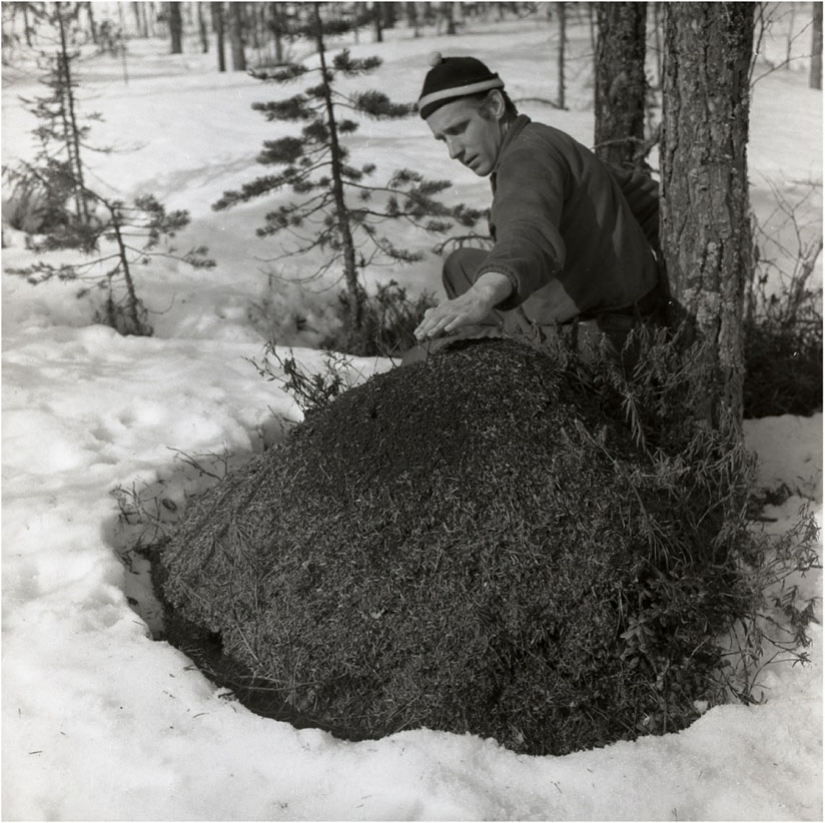
Gathering formic acid on the skin from an anthill in spring, Hälsingland, Sweden, 29 April 1956 (Photo Hilding Michelsson, Courtesy Hälsingslands Museum)

The anthills have been important parts of Swedish folk prescriptions. Records of their various therapeutic functions in Swedish folk medicine include a citation from the province of Småland in the 1740s that anthills were used for divination. The “first” was important in local folk medicine and lore [36]. For example, if a person put a stick in the first anthill found in spring and if ants crawled right up to the top of the stick, that person would not die during the year. Ants on the stick were also seen as a good prevention against diseases. To reap those benefits, people squeezed the ants and sucked the “juice”, i.e. formic acid [37]. One way to stay healthy throughout the year was to visit an anthill early in the spring, stir it and inhale the smoke of formic acid the irritated ants sprayed [38]. The first ants observed leaving the nest in spring were used to treat various skin diseases, for instance scabies. Low back pain was believed to be cured by throwing a handful of ants on the victim. The reasoning behind this treatment was that as the ant has a curved back, so *similia similibus*, i.e. “let likes be cured by likes” [16, 22]. More rational was the use of formic acid to cure warts [22] **(**Fig. 1**).**

Entire anthills were commonly cooked in remedies folk medicine all over Sweden. The water left after the ant nest had been boiled was used to wash the body for rheumatism [22, 39]. These types of baths have been common all over Sweden [38–40]. Still in the nineteenth century, “ant bath” (*myrbad*) instead of herbal bath was ordinated as a rheumatism treatment at spa institutions or certain ant bath houses in Sweden. Such baths are mentioned in the sources already in 1697. The bath was prepared by an addition of a decoction of ants, later also by an addition of formic acid tincture [41, 42]. In neighbouring Norway, rashes were treated using the same cure [43, 44]. Archives also record folk uses including the use of ant nests for magic [22, 39]. Despite the nests being used for different causes, disturbing or damaging an anthill was considered potentially dangerous and could cause rheumatic pain. In order to heal, the sufferer had to bathe in hot water prepared by boiling an anthill [39]. These treatments were documented also from Denmark, Finland, Norway and elsewhere in Europe [23, 44–47].

According to Carl Linnaeus, ant-based medicine was used against paralysis [17]. The use of ants as medicine against bad colds and paralysis seems to have been widespread [47–50]. However, products of the red wood ant were also important in folk medicine and used across the country.

A modern way to consume formic acid known already in the 1960s and still mentioned on social media is to let the irritated red wood ants spray their acid on an open sandwich held over the anthill [51]. This way of flavouring your sandwich is also mentioned in Swedish novels [52]. Ant vinegar was once considered a nice condiment in Norway and was made by tumbling ants into a pot of hot water, where they emitted a vinegar-like substance [46]. A similar kind of vinegar is also mentioned from the Swedish province Småland, where it was considered good against headache [53].

### The use of ant spirit

The use of spices and various botanicals to flavour schnapps is a part of the Swedes’ relationship with alcohol [54]. Usually, plants with medicinal virtues were used, for instance St. John’s worth, *Hypericum perforatum* L.; wormwood, *Artemisia absinthium* L.; caraway, *Carum carvi* L.; bog-myrtle, *Myrica gale* L*.*; etc. [55, 56]. Ants were also used for this purpose. Live ants were placed in a bottle, topped with plain *brännvin*, i.e. liquor distilled from grain or potatoes and left to infuse for several weeks. This made schnapps used for medicinal purposes, but was also considered good to drink [18]. This drink is mentioned by the eighteenth-century poet Carl-Mikael Bellman [57]. In his economic dictionary from 1781, Johan Fischerström has a long entry about home-distilled liquor, and he mentions among various berries, fruits, herbs and roots and also ants and anthills as useful to produce brännvin. He also suggests that the ant spirit could compete with many foreign and imported aquavits [58]*.* Pharmacist Franz Joachim von Aken suggests that the peasantry should distil liquor from a brew made of anthill and juniper berries. It would produce healthy liquor that could be made in springtime and in autumn [59].

Distilled liquor flavoured with ants was regarded as very effective against gout and rheumatism, certified by a woman from the province of Ångermanland. “It was good against all kind of diseases”, she continued [40]. It seems to have been a common home remedy all over Sweden in the past, and it was also ordinated by local healers [60–63]. Ant spirits was mentioned as a pharmaceutical product already in 1698. The acid oil (oleum) was sold in the pharmacies [64]. It was a common homemade remedy among the peasantry in Sweden [22, 60]. In the early twentieth century, it was still recommended as a cure for hunting dogs with rheumatism [65].

Homemade ant schnapps (Swedish *myrbrännvin*) is an alcohol (aquavit) that has been flavoured with formic acid, and was originally made as a remedy for a variety of symptoms and illness. The drink has a long tradition is still appreciated as flavoured schnapps by some people [66].

**Fig. 2:**
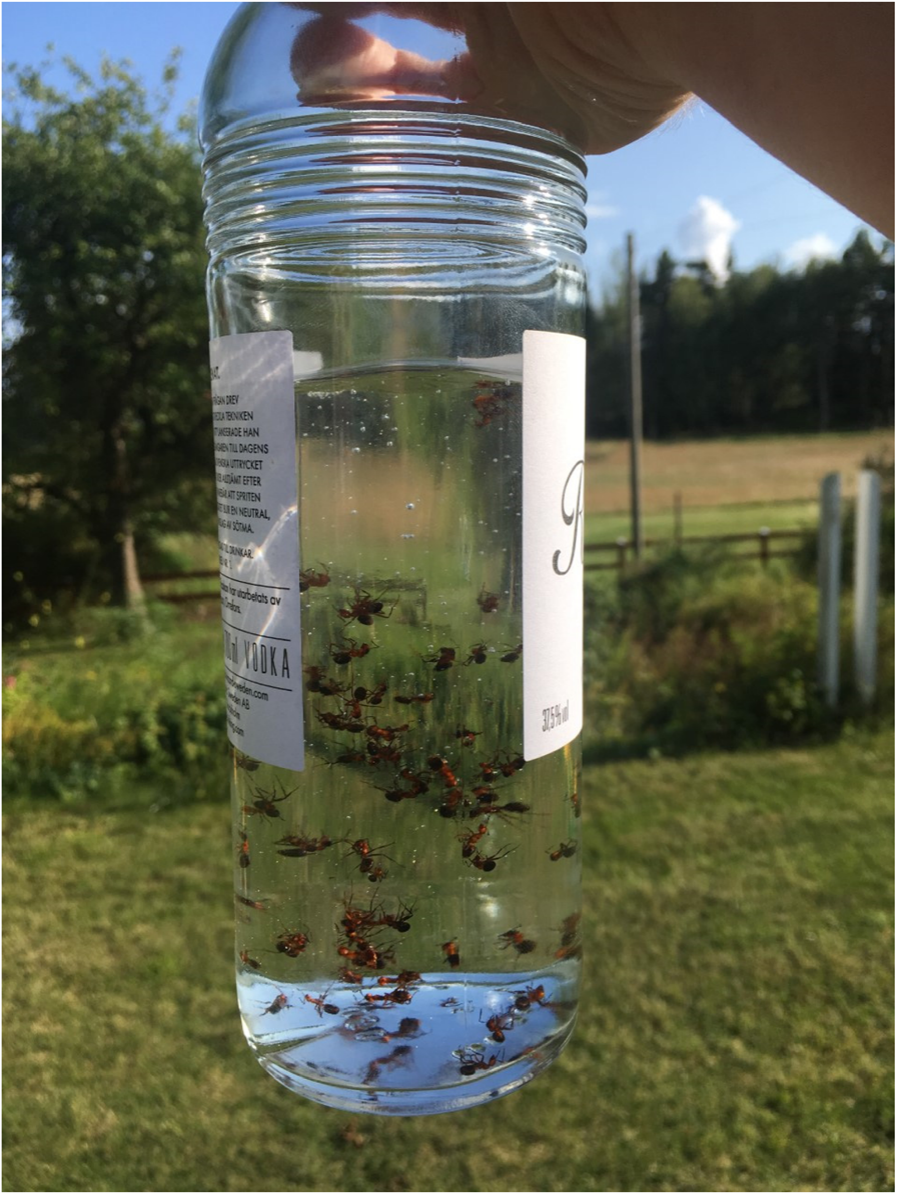
Homemade ant schnapps; vodka infused by red forest ant (Photo Isak Lidström, 2019)

The subject ant-flavoured liquor is still sometimes mentioned in newspaper reports from the countryside where supposedly old customs survive. A story from the Stockholm archipelago tells how a man a generation ago cured rheumatism and rash on the neck with ant liquor [67]. However, contemporary making of ant schnapps is at the most a hobby to flavour one’s own liquor. The interest for flavouring alcoholic beverages is a relatively common hobby, especially among men [55, 67]. Most popular is making bitter using wild harvested or garden St. John’s wort, *Hypericum maculatum*, and *H. perforatum*. Recipes are readily available not only in newspapers during spring and in social media, but also in handbooks. On Facebook and other social media, there are also plenty of recipes for making ant schnapps [68]. To make ant schnapps according to old customs is very simple. What is needed is a bottle of Swedish unflavoured brännvin (distilled liquor) and access to an anthill. A contemporary recipe is given in a cookbook from 2004: “Ant schnapps: one bottle of Renat (vodka), one tbsp. honey, 75 red wood ands. Find a decent sized anthill. Lick on a straw or a stick and put it on top of the hill. Shake off the ants in a bottle. When you get home you pour on vodka, honey and let it all sit for a day. The ants can be sieved off or left in the bottle” [69] (Fig. 2).

**Fig. 3:**
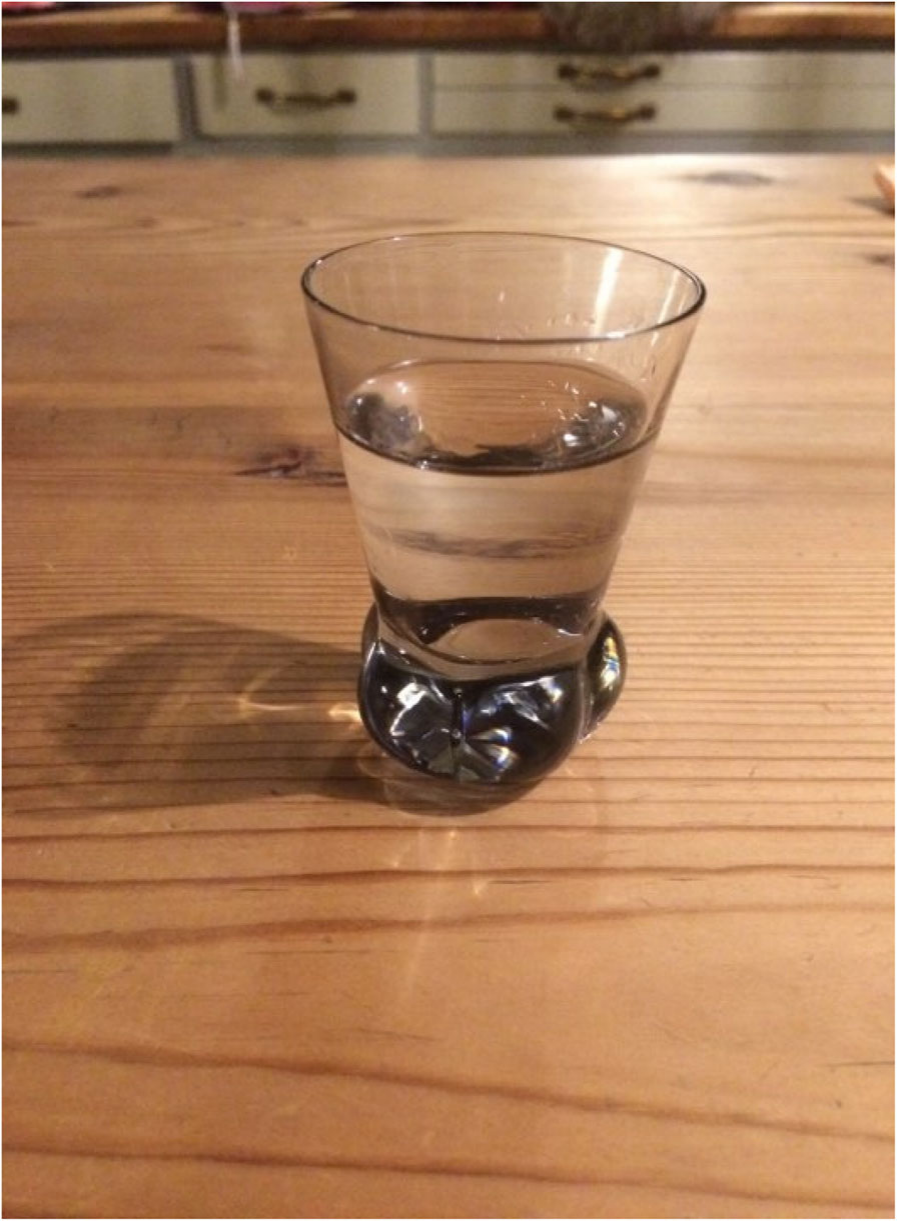
The homemade ant schnapps is usually served in a typical shot glass. It its drunk chilled. Many people sing a schnapps song before downing the shot of schnapps. The song ends with a toast “skål” (Photo Gabriel Lidström, 2019)

It is currently not illegal to harvest ants or removing parts of anthills in Sweden. Swedes are according to the law “every-man’s right” allowed to roam in the forests to gather berries, mushrooms and herbs without asking the landowner for permission. It is hard to say how many make ant schnapps today, but it is a living tradition. Aquavit, i.e. flavoured hard liquor, is traditionally consumed in a small shot glass (Swedish *nubbe*) to a traditional Swedish meal (especially with Midsummer eve’s pickled herring; at cray fish parties in August; for Christmas Eve dinner; or at a traditional Swedish buffet-style smorgasbord) or other meals as schnapps. The ant schnapps is today probably consumed mostly in connection with meals **(**Fig. 3**)**.

With a rise in interest to use insects as human food, some chefs have discovered ant schnapps and serve this together with food. With an increased interest to develop local ingredients in the New Nordic Cuisine, ant-flavoured alcohol has attracted a new attention. Gin flavoured with ants is recently highlighted as an interesting drink by Nordic chefs. Nordic Food Lab in Copenhagen, Denmark, has in 2013 together with The Cambridge Distillery produced a gin labelled Anty-Gin, spiced with red wood ants [70].

The use of red wood ants through history shows that the species has been an important provider of cultural ecosystem services. Predominantly, the ants and the formic acid they produce have been used for different medicinal purposes. These traditions have largely disappeared in Sweden and Scandinavia. One tradition remains today and this is the flavouring of aquavit with formic acid. Recently, this traditional use had been rediscovered by the supporters of the New Nordic Cuisine as an interesting way of using natural resources produced by insects [66, 69].

Decoction of anthill probably began with ancient folk medicine. Spirits seasoned with live ants are known at least from the seventeenth century. Formic acid and ant oil have also been available in the pharmacy, until the end of the nineteenth century. “Ant medicine” has been used especially for rheumatism and back pain. The idea behind this is probably associated with the crooked appearance of the ant. At first sight, it seems to have a crooked back. The tradition of using ants to heal should therefore be understood in the context of the idea of “let like be cured by like” [22].

## Conclusion

A convenient way of preparing ants for medicinal purposes was to make homemade ant schnapps. Making a medicament by seasoning aquavit with a certain proportion of ants to spirits dates back to the seventeenth century. This common practice of seasoning made the alcohol drinkable. Different spices could be added to contribute to specific healing properties, but it also gave good flavour to the spirits to increase its appreciation as a drink. Ant-flavoured aquavit has been used as a drink since at least the eighteenth century.

Over the past few decades, ant schnapps has been produced by people who have home cooking of aquavit as a hobby. There are many Swedes who, despite a great variety of commercial flavoured products, like to season their own aquavit. It is appreciated as a tasty and interesting drink and is in line with recently increased interest of food products made from insects.

## Data Availability

The data supporting the conclusions of this article are included within the article.
